# Safety and effectiveness of edoxaban in Japanese patients with nonvalvular atrial fibrillation: Final report of a two‐year postmarketing surveillance study (ETNA‐AF‐Japan)

**DOI:** 10.1002/joa3.12520

**Published:** 2021-02-24

**Authors:** Takeshi Yamashita, Yukihiro Koretsune, Tomoko Nagao, Kazuhito Shiosakai

**Affiliations:** ^1^ The Cardiovascular Institute Tokyo Japan; ^2^ National Hospital Organization Osaka National Hospital Osaka Japan; ^3^ Pharmacoepidemiology & PMS Department Daiichi Sankyo Co. Ltd Tokyo Japan; ^4^ Data Intelligence Department Daiichi Sankyo Co. Ltd Tokyo Japan

**Keywords:** anticoagulant, atrial fibrillation, direct‐acting oral anticoagulants, edoxaban, postmarketing product surveillance

## Abstract

**Background:**

Direct oral anticoagulants (DOACs) are the recommended first‐line therapy for ischemic stroke prevention in patients with nonvalvular atrial fibrillation (NVAF). However, the safety and effectiveness of edoxaban for this indication requires monitoring over the long term in real‐world settings.

**Methods:**

ETNA‐AF‐Japan (trial no. UMIN000017011) was a prospective, multicenter observational study (part of postmarketing surveillance in Japan). NVAF patients due to receive edoxaban to prevent ischemic stroke were enrolled between 13 April 2015 and 30 September 2017.

**Results:**

A total of 11 569 patients were enrolled. For the 11 111 patients (female, 40.6%) whose data comprised the safety analysis set, age, body weight, creatinine clearance (CLcr), and CHADS_2_ score were 74.2 ± 10.0 years, 60.0 ± 12.7 kg, 63.9 ± 25.8 mL/min, and 2.2 ± 1.3, respectively (mean ± SD). The majority (86.3%) received edoxaban in accordance with package insert information. The mean duration of treatment was 561.9 ± 261.2 days. The annual incidence (95% confidence interval) of all bleeding events and major bleeding events was 5.60% (5.25%–5.98%) and 1.02% (0.88%–1.18%), respectively. The annual incidence of ischemic stroke (excluding transient ischemic attack, TIA) or systemic embolism was 1.08% (0.93%–1.25%). Multivariate analysis showed low body weight, low CLcr, history of gastrointestinal bleeding, anemia, and use of an antiplatelet agent to be associated with major bleeding, and history of ischemic stroke or TIA, vascular disease, and antiplatelet agent use to be associated with ischemic stroke (excluding TIA) or systemic embolism.

**Conclusions:**

These results provide real‐world evidence for the long‐term good safety and effectiveness profile of edoxaban in Japanese NVAF patients under clinical practice.

## Graphical abstract

This prospective, multicenter observational study was carried out to evaluate the safety and effectiveness of edoxaban as first‐line therapy to prevent ischemic stroke in Japanese patients with nonvalvular atrial fibrillation (NVAF). Annual incidence (95% confidence interval) of all bleeding events, major bleeding events, and ischemic stroke (excluding transient ischemic attack, TIA) or systemic embolism was 5.60% (5.25%–5.98%), 1.02% (0.88%–1.18%), and 1.08% (0.93%–1.25%), respectively (safety analysis set, 11 111; effectiveness analysis set, 11 063; mean duration of treatment, 561.9 ± 261.2 days). These results add to the evidence for the long‐term good safety and effectiveness profile of edoxaban in Japanese patients with NVAF in a real‐world clinical setting.

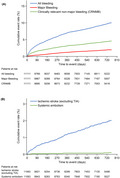

## 1 INTRODUCTION

The results of randomized clinical trials have shown the advantages of direct oral anticoagulants (DOACs) over warfarin for preventing stroke in patients with atrial fibrillation (AF).^1^, ^2^, ^3^, ^4^ Consequently, the Japanese Circulation Society guidelines,^5^ the Japanese Circulation Society–Japanese Heart Rhythm Society joint guidelines,^6^ and the European Heart Rhythm Association guidelines^7^ now recommend DOACs in preference to warfarin as first‐line anticoagulant therapy for ischemic stroke prevention in patients with nonvalvular atrial fibrillation (NVAF). However, the guidelines state that clinical experience of treatment with these newer drugs needs to be recorded and their performance monitored over the long term to ensure that they are acceptable in terms of the balance between risk and benefit; their safety profile is a particular concern, because AF patients commonly have risk factors such as concomitant diseases.

The DOAC edoxaban, taken once daily, directly and reversibly inhibits factor Xa. It is indicated for the prevention of ischemic stroke and systemic embolism in NVAF patients.^8^, ^9^, ^10^, ^11^ Two formulations are available: tablet and orally disintegrating. The latter is particularly useful for elderly patients who might otherwise have difficulty swallowing the tablets.

Confirmation of the efficacy and acceptable safety profile of edoxaban has been provided by the results of a pivotal confirmatory phase 3 study: ENGAGE AF‐TIMI 48 (Effective aNticoaGulation with factor xA next GEneration in Atrial Fibrillation—Thrombolysis In Myocardial Infarction 48).^4^ The inclusion and exclusion criteria for this randomized controlled trial were strict, therefore the study population did not include all potential beneficiaries of the treatment. Therefore, an investigation of the safety and effectiveness of long‐term edoxaban use in a real‐world clinical setting was necessary. ETNA‐AF‐Japan (Edoxaban Treatment in routiNe clinical prActice in patients with nonvalvular Atrial Fibrillation; trial no. UMIN000017011) was initiated to evaluate the occurrence of ischemic stroke/systemic embolism and bleeding episodes in ≥10 000 patients over 2 years. The findings of ETNA‐AF‐Japan will help determine how best to treat patients, particularly elderly patients, with AF. The findings are expected to be especially useful in Japan, where more than 700 000 people are estimated to have AF and over a quarter of the population is ≥65 years old (according to the 2015 census).^12^, ^13^

We reported the 3‐month^14^ and 1‐year^15^ interim analysis results from ETNA‐AF‐Japan in 2019 and 2020, respectively. These articles included data on patient demographics, clinical characteristics, dosing status, and edoxaban's safety and effectiveness. Here, we report the final results of ETNA‐AF‐Japan, including the results of analyses conducted to identify factors associated with the drug's safety and effectiveness.

## 2 METHODS

### 2.1 Study design

ETNA‐AF‐Japan is a real‐world, prospective, multicenter observational study and part of postmarketing surveillance. Our aim in carrying out this study was to evaluate edoxaban's safety and effectiveness in Japanese NVAF patients by analyzing baseline and clinical data collected over 2 years. ETNA‐AF‐Japan was in accordance with the Good Post‐marketing Study Practice of the Ministry of Health, Labor, and Welfare of Japan (MHLW). Methodological details are available in the report of the results of interim analyses based on 3‐month data.14

### 2.2 Patient population

The target number of study participants was 10 000. Adult NVAF patients for whom edoxaban had been prescribed for prevention of ischemic stroke and systemic embolism were eligible, on the condition that they had not received the drug before. Additional eligibility criteria were the patients’ ability to start treatment during enrollment, their availability for long‐term follow‐up, and their agreement to provide, at the time of registration, written informed consent to participate.

### 2.3 Study variables

The patients' baseline and clinical data, including information on medication status (for edoxaban and concomitant drugs), medical history and comorbidities, and details of nonpharmacological treatments for AF or surgical procedures, were recorded in case report forms by their physicians. The baseline data were used to calculate HAS‐BLED,^16^ CHADS_2_,^17^ and CHA_2_DS_2_‐VASc^18^ scores. The HAS‐BLED scores were based on a number of risk factors (namely hypertension, abnormal renal or liver function, stroke, bleeding, elderly status, and drug use, but not international normalized ratio or alcohol use).

The following events were recorded: death, stroke other than transient ischemic attack (TIA), systemic embolism, myocardial infarction, and adverse events (AEs), including bleeding events.

### 2.4 Initial daily dose and administration

As specified on the Japanese package insert for edoxaban, the recommended oral daily dose is 60 and 30 mg for adult patients with body weight >60 kg and ≤60 kg, respectively.^11^ The lower dose is also recommended for patients with either renal dysfunction (creatinine clearance, CLcr, ≤50 mL/min) or concomitant use of a P‐glycoprotein inhibitor (erythromycin, cyclosporine, quinidine, or verapamil), or both.

### 2.5 Study outcomes

The study outcomes were AEs, including bleeding events, for evaluation of safety, and clinical events, including death, ischemic stroke (excluding TIA), systemic embolism, and myocardial infarction, for evaluation of effectiveness. Physicians categorized bleeding as major bleeding, clinically relevant nonmajor bleeding (CRNMB), or minor bleeding, according to definitions in the report of the ENGAGE AF‐TIMI 48 study, with minor modifications.^4^, ^15^

### 2.6 Statistical analysis

To estimate the cumulative incidence of bleeding events from the start of treatment to the date of the last dose, the Kaplan–Meier method was used. To determine factors associated with bleeding events during this period, variables identified as significant (*P* < .05) in a univariate analysis (Cox proportional hazards model) were used as candidate variables in the forward‐backward stepwise method, and selected factors were included in the subsequent multivariate analysis using the same model. Age was compulsorily included in the multivariate analysis. For hypothesis testing, the significance level was set at 5%, and the confidence intervals for both sides were set at 95%. Missing data were not computed. For all analyses, SAS System Release 9.2 (SAS Institute Inc, Cary, NC, USA) was used.

## 3 RESULTS

### 3.1 Patient characteristics at baseline and patient disposition

**FIGURE 1:**
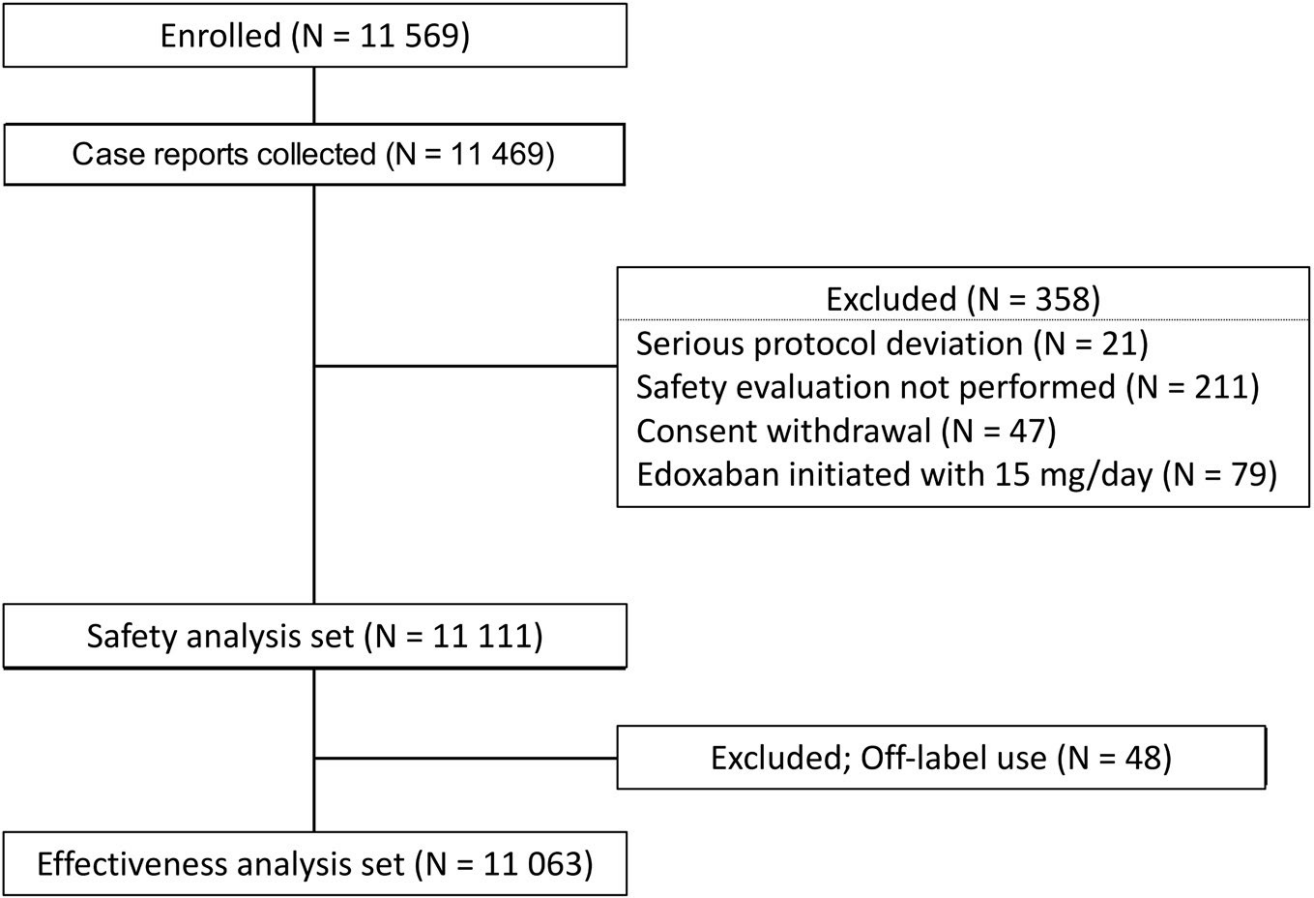
The ETNA‐AF‐Japan study: patient disposition

A total of 11 569 patients were enrolled in 1367 Japanese centers in the period from 13 April 2015 to 30 September 2017. Of the patents whose case reports were collected, data from 358 were excluded for the reasons of serious deviation for the protocol, safety evaluation not carried out, withdrawal of consent, and edoxaban prescribed at an initial dose of 15 mg/day; consequently, data from 11 111 patients were included in the safety analysis. After the exclusion of 48 patients for the reason of off‐label use, data from 11 063 patients were included in the effectiveness analysis (Figure 1).

Patient characteristics at baseline are summarized in Table 1. Of the 11 111 patients whose data comprised the safety analysis set, mean age, body weight, CLcr, and CHADS_2_ score were 74.2 ± 10.0 years, 60.0 ± 12.7 kg, 63.9 ± 25.8 mL/min, and 2.2 ± 1.3, respectively. Regarding the starting daily dose of edoxaban, 251 received an over‐dose (ie 60 mg/day despite fulfilling the dose reduction criteria), 2750 received the recommended standard dose (60 mg/day), 6645 received the recommended reduced dose (30 mg/day), and 1235 received an under‐dose (ie, 30 mg/day despite not fulfilling the dose reduction criteria). Mean age, CHADS_2_ score, and HAS‐BLED score showed a tendency to be higher in patients receiving edoxaban at the recommended reduced dose (77.4 years, 2.3 and 2.1) than the standard dose (67.4 years, 1.8 and 1.7). The trend was similar in the under‐dose group (72.9 years, 2.2 and 2.1).

**TABLE 1:** Baseline characteristics of patients in the ETNA‐AF‐Japan study^a^

Variable	All (N = 11 111)	Daily dose at the start of the study (N = 10 881)			
		Over‐dose (60 mg) (N = 251)	Recommended standard dose (60 mg) (N = 2750)	Recommended reduced dose (30 mg) (N = 6645)	Under‐dose (30 mg) (N = 1235)
Female	4510 (40.6)	87 (34.7)	337 (12.3)	3775 (56.8)	218 (17.7)
Age, years					
Mean ± SD	74.2 ± 10.0	70.1 ± 10.8	67.4 ± 9.3	77.4 ± 8.8	72.9 ± 9.2
≥75	5827 (52.4)	97 (38.6)	620 (22.5)	4380 (65.9)	612 (49.6)
Body weight, kg					
Mean ± SD	60.0 ± 12.7	60.2 ± 8.4	72.5 ± 10.0	52.8 ± 8.6	69.9 ± 8.0
≤60	6035 (54.3)	169 (67.3)	0 (0.0)	5866 (88.3)	0 (0.0)
Creatinine clearance, mL/min^b^					
Mean ± SD	63.9 ± 25.8	67.4 ± 25.5	86.4 ± 25.2	52.5 ± 18.9	73.7 ± 21.3
<30	538 (4.8)	1 (0.4)	0 (0.0)	537 (8.1)	0 (0.0)
30–50	2928 (26.4)	68 (27.1)	0 (0.0)	2860 (43.0)	0 (0.0)
>50 to <80	4982 (44.8)	117 (46.6)	1293 (47.0)	2702 (40.7)	870 (70.4)
≥80	2372 (21.3)	63 (25.1)	1457 (53.0)	487 (7.3)	365 (29.6)
Unknown	291 (2.6)	2 (0.8)	0 (0.0)	59 (0.9)	0 (0.0)
Type of atrial fibrillation					
Paroxysmal	5123 (46.1)	127 (50.6)	1266 (46.0)	3099 (46.6)	522 (42.3)
Persistent (>7 days)	4265 (38.4)	98 (39.0)	1106 (40.2)	2488 (37.4)	482 (39.0)
Permanent	1707 (15.4)	25 (10.0)	373 (13.6)	1051 (15.8)	230 (18.6)
Unknown	16 (0.1)	1 (0.4)	5 (0.2)	7 (0.1)	1 (0.1)
CHADS_2_ score					
Mean ± SD	2.2 ± 1.3	1.9 ± 1.4	1.8 ± 1.2	2.3 ± 1.4	2.2 ± 1.4
CHA_2_DS_2_‐VASc score					
Mean ± SD	3.5 ± 1.6	3.0 ± 1.6	2.7 ± 1.5	3.9 ± 1.6	3.3 ± 1.6
HAS‐BLED score^c^					
Mean ± SD	2.0 ± 1.0	1.8 ± 1.0	1.7 ± 1.0	2.1 ± 0.9	2.1 ± 1.0
Switch from other anticoagulants					
Total^d^	2526 (22.7)	46 (18.3)	631 (22.9)	1513 (22.8)	311 (25.2)
Warfarin	1238 (11.1)	13 (5.2)	303 (11.0)	746 (11.2)	167 (13.5)
Rivaroxaban	445 (4.0)	10 (4.0)	123 (4.5)	261 (3.9)	47 (3.8)
Apixaban	362 (3.3)	8 (3.2)	82 (3.0)	219 (3.3)	45 (3.6)
Dabigatran	316 (2.8)	6 (2.4)	89 (3.2)	177 (2.7)	40 (3.2)
Others	166 (1.5)	10 (4.0)	34 (1.2)	110 (1.7)	12 (1.0)
Bleeding history					
Intracranial bleeding	258 (2.3)	3 (1.2)	53 (1.9)	168 (2.5)	33 (2.7)
Gastrointestinal bleeding	175 (1.6)	3 (1.2)	38 (1.4)	105 (1.6)	26 (2.1)
Medical history and comorbidities					
Hypertension	8004 (72.0)	165 (65.7)	2035 (74.0)	4722 (71.1)	948 (76.8)
Diabetes mellitus	2592 (23.3)	56 (22.3)	736 (26.8)	1393 (21.0)	364 (29.5)
Dyslipidemia	4036 (36.3)	77 (30.7)	1145 (41.6)	2238 (33.7)	513 (41.5)
Myocardial infarction	426 (3.8)	6 (2.4)	80 (2.9)	270 (4.1)	65 (5.3)
Angina pectoris	1210 (10.9)	29 (11.6)	247 (9.0)	725 (10.9)	191 (15.5)
Heart failure/left ventricular systolic dysfunction	3016 (27.1)	56 (22.3)	545 (19.8)	2052 (30.9)	322 (26.1)
Ischemic stroke/transient ischemic attack	2291 (20.6)	50 (19.9)	512 (18.6)	1458 (21.9)	234 (18.9)
Cancer	867 (7.8)	24 (9.6)	169 (6.1)	564 (8.5)	103 (8.3)
Gastric ulcer	410 (3.7)	7 (2.8)	96 (3.5)	261 (3.9)	42 (3.4)
Anemia	458 (4.1)	11 (4.4)	43 (1.6)	358 (5.4)	39 (3.2)

^a^ A total of 230 patients for whom dose adjustment factors were unknown were excluded;

^b^ Creatinine clearance was estimated using the Cockcroft & Gault equation;

^c^ Neither labile international normalized ratio nor alcohol use were counted; thus, the highest total score was seven.

^d^ Some overlap present.

### 3.2 Medication status

**FIGURE 2:**
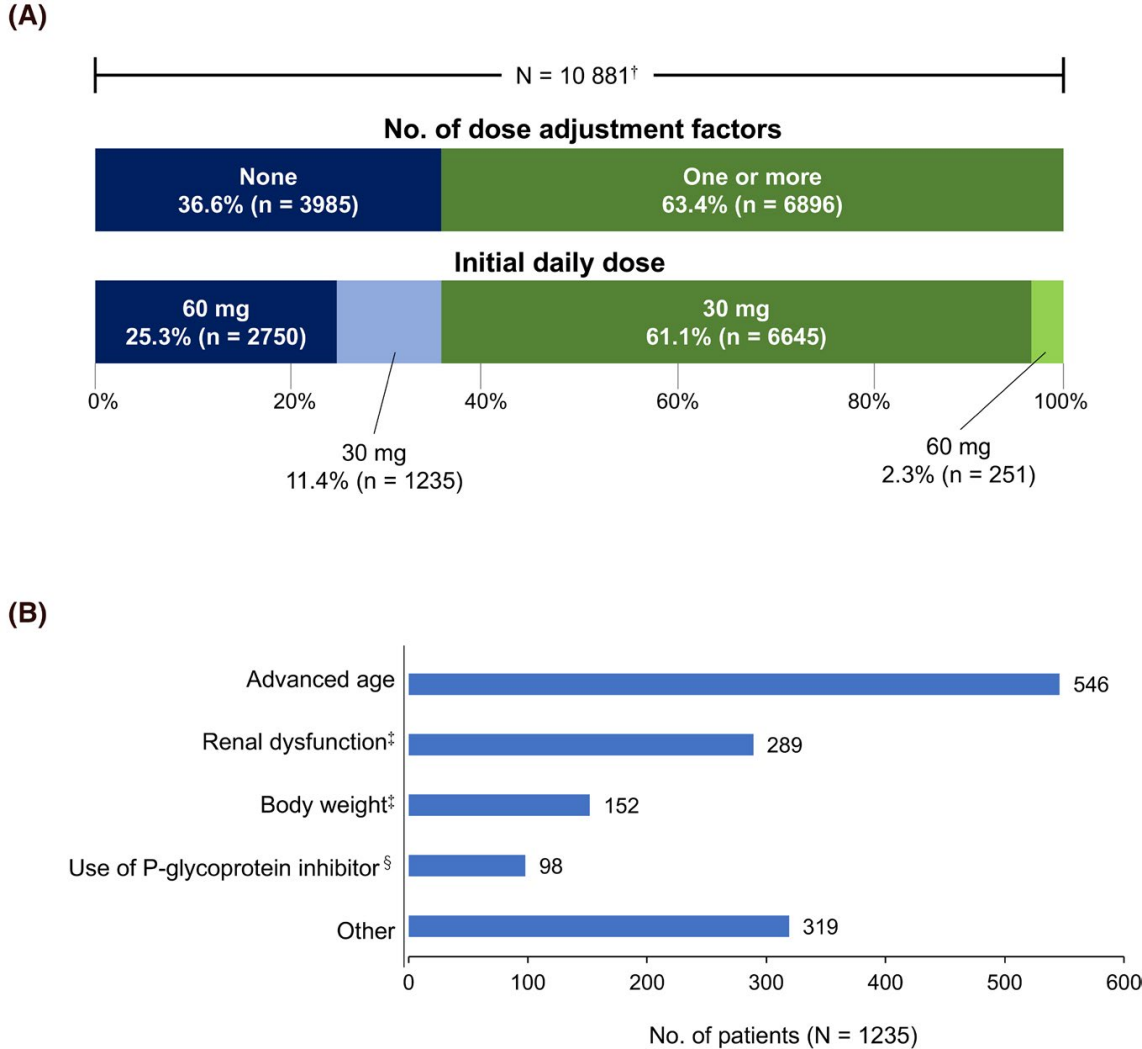
Edoxaban dosing status for patients in the ETNA‐AF‐Japan study. A, Number of dose adjustment factors and initial daily dose. B, Reasons given by physicians for prescribing the under‐dose (30 mg) for patients with no dose adjustment factors. In some cases, multiple reasons were given. ^†^The number of patients whose data comprised the safety analysis set (N = 11 111) minus the number of patients for whom data on dose adjustment factors were unavailable (n = 230). ^‡^Did not meet the criteria for dose reduction as stated on the package insert. ^§^Other than quinidine, verapamil, erythromycin, and cyclosporine

At the start of treatment, 6896 (63.4%) of the 10 881 patients with available data on dose adjustment factors had one or more dose adjustment factors (Figure 2A). Nearly 90% (9395/10 881, 86.3%) of patients received edoxaban in accordance with the instructions on the package insert. However, 1235 patients without dose adjustment factors received the under‐dose, 30 mg, and 251 patients with one or more dose adjustment factors received the over‐dose, 60 mg. The most common reason given by physicians for prescribing the under‐dose was old age, followed by renal dysfunction (Figure 2B). Other reasons included those related to patients’ bleeding risk, for example, history of bleeding or antithrombotic agent use.

Edoxaban treatment status is summarized in Table 2. At 2 years after treatment started, 7753 patients were continuing to receive edoxaban, and 3358 have completed or discontinued the drug; thus, the treatment continuation rate at 2 years was 69.8% (7753/11 111). Regarding adherence, 94.6% of patients (10 513/11 111) took their medication every day. Mean ± SD and median treatment duration were 561.9 ± 261.2 days and 719.0 days, respectively. The most common reason for completing or discontinuing treatment was patients' failure to visit the hospital or their transfer to another hospital (11.7%), followed by the occurrence of AEs or clinical events (9.2%) and treatment completed as planned (4.7%).

**TABLE 2:** Edoxaban treatment status of patients whose data comprised the safety analysis set (N = 11 111)

Treatment status	n (%)
Total	
Ongoing treatment with edoxaban	7753 (69.8)
Completion or discontinuation of treatment with edoxaban	3358 (30.2)
Reasons for completion or discontinuation of treatment with edoxaban^a^	
Failure to visit the hospital or transfer to a different hospital	1302 (11.7)
Occurrence of adverse events or clinical events	1024 (9.2)
Treatment completed as planned	518 (4.7)
Switch to other drugs	416 (3.7)
Plan to receive nonpharmacological therapy for atrial fibrillation	35 (0.3)
Plan to receive an invasive procedure (including minor surgery)	30 (0.3)

^a^ In some cases, more than one reason applied.

### 3.3 Safety analysis

**FIGURE 3:**
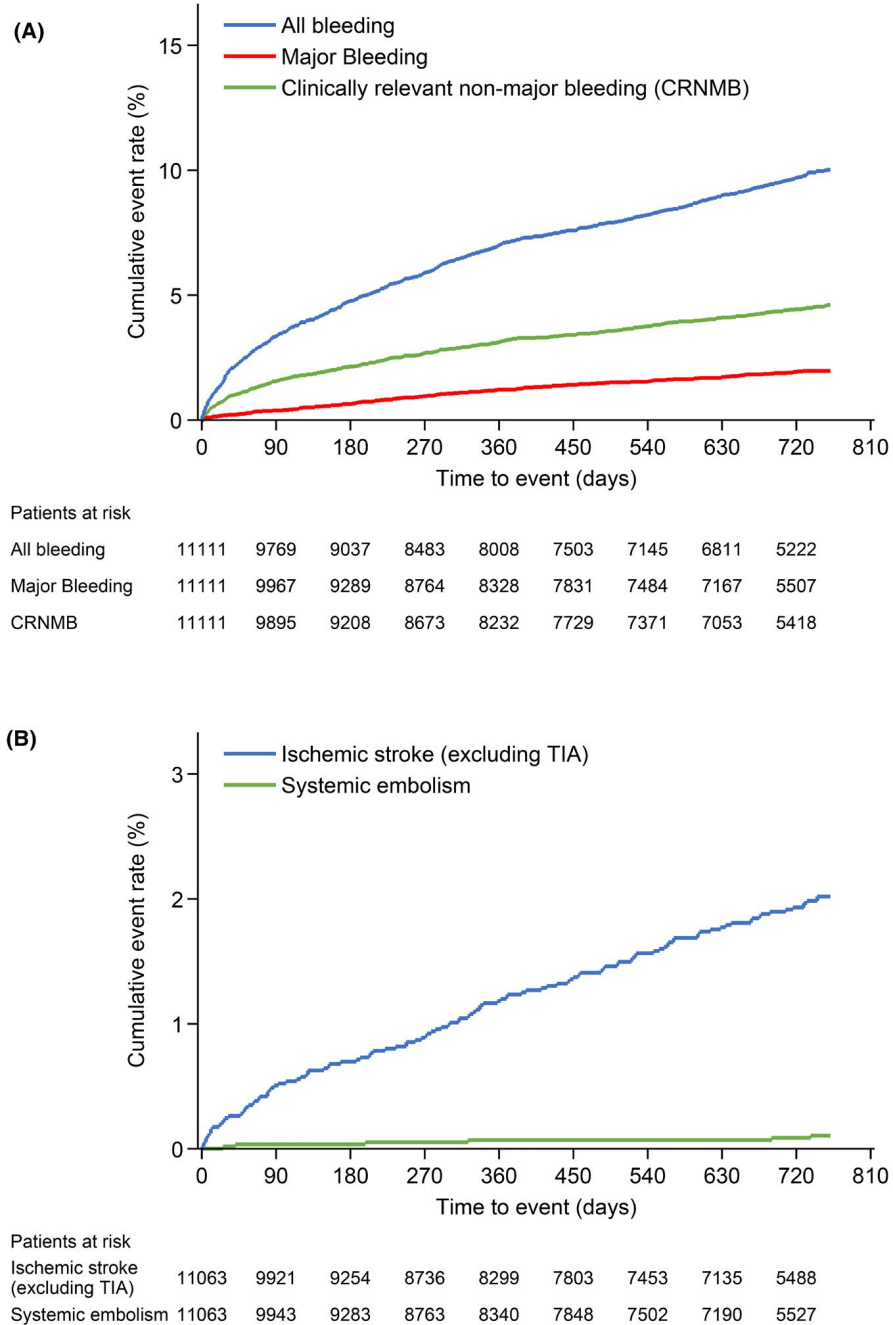
Cumulative incidence of bleeding events (A) and clinical events (B) in patients with nonvalvular atrial fibrillation who received edoxaban for ≤2 years. CRNMB, clinically relevant nonmajor bleeding; TIA, transient ischemic attack

Regarding bleeding events, their cumulative incidence increased over 2 years (Figure 3A). Annual incidence of all bleeding events, major bleeding, CRNMB, and minor bleeding was 5.60%, 1.02%, 2.48%, and 2.27%, respectively (Table 3; full details of the 183 major bleeding events recorded for 173 patients are available in Table S2). The annual incidence of all bleeding was 6.00% and 5.76% in the recommended standard dose (60 mg/day) group and the recommended reduced dose (30 mg/day) group, respectively. The annual incidence of major bleeding was 0.75% and 1.21% in the recommended standard dose (60 mg/day) group and recommended reduced dose (30 mg/day) group, respectively.

**TABLE 3:** Incidence of outcome events

Bleeding events	Total (N = 11 111)	Daily dose at the start of the study (N = 10 881^a^)			
		Over‐dose (60 mg) (N = 251)	Recommended standard dose (60 mg) (N = 2750)	Recommended reduced dose (30 mg) (N = 6645)	Under‐dose (30 mg) (N = 1235)
All Bleeding					
n (%)	921 (8.29)	15 (5.98)	246 (8.95)	562 (8.46)	87 (7.04)
Annual incidence, % (95% CI)	5.60 (5.25‐5.98)	4.67 (2.82‐7.75)	6.00 (5.30‐6.80)	5.76 (5.30‐6.25)	4.53 (3.67‐5.59)
Major bleeding					
n (%)	173 (1.56)	1 (0.40)	32 (1.16)	122 (1.84)	17 (1.38)
Annual incidence, % (95% CI)	1.02 (0.88‐1.18)	0.30 (0.04‐2.12)	0.75 (0.53‐1.05)	1.21 (1.01‐1.45)	0.86 (0.54‐1.39)
Cerebral hemorrhage					
n (%)	56 (0.50)	0 (0.00)	13 (0.47)	39 (0.59)	3 (0.24)
Annual incidence, % (95% CI)	0.33 (0.25‐0.43)	—	0.30 (0.18‐0.52)	0.39 (0.28‐0.53)	0.15 (0.05‐0.47)
Gastrointestinal bleeding					
n (%)	80 (0.72)	1 (0.40)	9 (0.33)	60 (0.90)	10 (0.81)
Annual incidence, % (95% CI)	0.47 (0.38‐0.58)	0.30 (0.04‐2.12)	0.21 (0.11‐0.40)	0.59 (0.46‐0.77)	0.51 (0.27‐0.94)
CRNMB					
n (%)	418 (3.76)	8 (3.19)	109 (3.96)	262 (3.94)	33 (2.67)
Annual incidence, % (95% CI)	2.48 (2.26‐2.73)	2.43 (1.21‐4.85)	2.58 (2.14‐3.11)	2.63 (2.33‐2.96)	1.68 (1.20‐2.37)
Minor bleeding					
n (%)	381 (3.43)	6 (2.39)	122 (4.44)	209 (3.15)	40 (3.24)
Annual incidence, % (95% CI)	2.27 (2.06‐2.51)	1.83 (0.82‐4.07)	2.91 (2.44‐3.48)	2.10 (1.83‐2.41)	2.05 (1.51‐2.80)

Clinical events	Total (N = 11 063)	Daily dose at the start of the study (N = 10 836^b^)			
		Over‐dose (60 mg) (N = 249)	Recommended standard dose (60 mg) (N = 2743)	Recommended reduced dose (30 mg) (N = 6612)	Under‐dose (30 mg) (N = 1232)
Ischemic stroke^c^ or systemic embolism					
n (%)	183 (1.65)	4 (1.61)	44 (1.60)	120 (1.81)	12 (0.97)
Annual incidence, % (95% CI)	1.08 (0.93‐1.25)	1.21 (0.45‐3.22)	1.03 (0.77‐1.38)	1.20 (1.00‐1.43)	0.61 (0.35‐1.07)
Ischemic stroke^c^					
n (%)	175 (1.58)	4 (1.61)	42 (1.53)	114 (1.72)	12 (0.97)
Annual incidence, % (95% CI)	1.03 (0.89‐1.20)	1.21 (0.45‐3.22)	0.98 (0.73‐1.33)	1.14 (0.95‐1.36)	0.61 (0.35‐1.07)
Systemic embolism					
n (%)	9 (0.08)	0 (0.00)	2 (0.07)	7 (0.11)	0 (0.00)
Annual incidence, % (95% CI)	0.05 (0.03‐0.10)	—	0.05 (0.01‐0.19)	0.07 (0.03‐0.15)	—
Stroke^c^ or systemic embolism					
n (%)	244 (2.21)	4 (1.61)	59 (2.15)	160 (2.42)	17 (1.38)
Annual incidence, % (95% CI)	1.44 (1.27‐1.63)	1.21 (0.45‐3.22)	1.38 (1.07‐1.78)	1.60 (1.37‐1.86)	0.86 (0.54‐1.39)
Stroke^c^					
n (%)	236 (2.13)	4 (1.61)	57 (2.08)	154 (2.33)	17 (1.38)
Annual incidence, % (95% CI)	1.39 (1.23‐1.58)	1.21 (0.45‐3.22)	1.33 (1.03‐1.73)	1.54 (1.31‐1.80)	0.86 (0.54‐1.39)
Cerebral hemorrhage					
n (%)	64 (0.58)	0 (0.00)	15 (0.55)	43 (0.65)	5 (0.41)
Annual incidence, % (95% CI)	0.38 (0.29‐0.48)	—	0.35 (0.21‐0.58)	0.43 (0.32‐0.58)	0.25 (0.11‐0.61)
Myocardial infarction					
n (%)	21 ((0.19)	0 (0.00)	3 (0.11)	13 (0.20)	3 (0.24)
Annual incidence, % (95% CI)	0.12 (0.08‐0.19)	—	0.07 (0.02‐0.22)	0.13 (0.07‐0.22)	0.15 (0.05‐0.47)
Death					
n (%)	218 (1.97)	6 (2.41)	26 (0.95)	169 (2.56)	12 (0.97)
Annual incidence, % (95% CI)	1.28 (1.12‐1.46)	1.81 (0.81‐4.03)	0.60 (0.41‐0.89)	1.67 (1.44‐1.95)	0.61 (0.34‐1.07)
Major cardiovascular events (MACE)					
n (%)	309 (2.79)	7 (2.81)	62 (2.26)	207 (3.13)	26 (2.11)
Annual incidence, % (95% CI)	1.83 (1.63‐2.04)	2.11 (1.01‐4.43)	1.45 (1.13‐1.86)	2.07 (1.80‐2.37)	1.32 (0.90‐1.94)
All events (combined events)^d^					
n (%)	461 (4.17)	10 (4.02)	83 (3.03)	327 (4.95)	32 (2.60)
Annual incidence, % (95% CI)	2.72 (2.49‐2.98)	3.02 (1.62‐5.61)	1.94 (1.57‐2.41)	3.26 (2.93‐3.64)	1.63 (1.15‐2.30)

Abbreviations: CI, confidence interval; CRNMB, clinically relevant non‐major bleeding.

^a^ The safety analysis set minus data from the 230 patients whose dose adjustment factors were unknown.

^b^ The effectiveness analysis set minus data from the 227 patients whose dose adjustment factors were unknown.

^c^ Excluding transient ischemic attack.

^d^ All events include death, stroke (excluding transient ischemic attack), systemic embolism, and myocardial infarction.

Multivariate analysis identified bodyweight, CLcr, prior gastrointestinal bleeding, anemia, and antiplatelet agent use as risk factors for major bleeding (*P* < .05) (Table 4). Table 5 shows edoxaban's safety and effectiveness when used at recommended doses in patients with specific backgrounds, for example, those who were not included in sufficient numbers in pivotal clinical trials. Compared with the total study population, the incidence of both all bleeding and major bleeding showed a tendency to be higher in each of the patient subgroups. Incidence of all bleeding showed a tendency to be higher in patients with prior bleeding (21.33%) and in older patients (≥80 years) with prior bleeding (24.32%) than in the total study population (8.60%). Incidence of major bleeding showed a tendency to be higher in older patients (≥80 years) with prior bleeding than in the total population (7.43% vs 1.64%).

**TABLE 4:** Factors associated with major bleeding and ischemic stroke or systemic embolism

Factor	Major bleeding				Ischemic stroke (excluding TIA) or systemic embolism			
	Univariate		Multivariate		Univariate		Multivariate	
	HR [95% CI]	*P*	Adjusted HR [95% CI]	*P*	HR [95% CI]	*P*	Adjusted HR [95% CI]	*P*
Female	0.909 [0.669, 1.235]	.54	—	—	0.793 [0.585, 1.075]	.13	—	—
Age, years								
<65	Reference	<.001	Reference	.56	Reference	<.01	Reference	.13
65 to <75	1.445 [0.780, 2.679]		1.027 [0.530, 1.989]		1.503 [0.848, 2.663]		1.224 [0.689, 2.174]	
≥75	2.424 [1.366, 4.301]		1.252 [0.637, 2.458]		2.082 [1.215, 3.566]		1.567 [0.910, 2.696]	
Body weight, kg								
>60	Reference	<.001	Reference	<.01	Reference	.07	—	
40‐60	1.054 [0.771, 1.442]		0.727 [0.514, 1.028]		1.042 [0.770, 1.408]		—	
<40	3.286 [1.854, 5.827]		1.797 [0.953, 3.386]		2.138 [1.107, 4.130]		—	
CLcr, mL/min								
≥80	Reference	<.0001	Reference	<.01	Reference	.01	—	
>50 to <80	1.821 [1.085, 3.057]		1.725 [0.964, 3.087]		1.151 [0.753, 1.759]		—	
30‐50	3.076 [1.828, 5.176]		2.590 [1.352, 4.958]		1.786 [1.159, 2.753]		—	
<30	4.856 [2.502, 9.423]		3.457 [1.536, 7.780]		1.890 [0.947, 3.772]		—	
Liver dysfunction^a^								
Normal	Reference	.49	—					
Mild	1.316 [0.693, 2.498]		—					
Moderate or severe	1.669 [0.532, 5.236]		—					
Initial dose of edoxaban								
Recommended dose	Reference	.32	—		Reference	.11	—	
Under‐dose	0.811 [0.491, 1.338]		—		0.536 [0.298, 0.963]		—	
Over‐dose	0.274 [0.038, 1.955]		—		1.026 [0.381, 2.767]		—	
Prior intracranial bleeding	2.207 [1.085, 4.488]	.03	—					
Prior gastrointestinal bleeding	3.396 [1.736, 6.643]	<.001	2.547 [1.277, 5.080]	<.01				
Hypertension	1.427 [0.985, 2.068]	.06	—		1.258 [0.889, 1.781]	.20	—	
Diabetes mellitus	1.365 [0.986, 1.889]	.06	—		1.402 [1.024, 1.920]	.04	—	
Heart failure or left ventricular systolic dysfunction	1.678 [1.233, 2.282]	<.01	—		0.962 [0.690, 1.340]	.82	—	
Ischemic stroke or TIA	1.732 [1.252, 2.398]	<.001	—		3.838 [2.872, 5.128]	<.0001	3.250 [2.411, 4.381]	<.0001
Cancer	1.718 [1.088, 2.711]	.02	—		1.438 [0.894, 2.312]	.13	—	
Gastric ulcer	2.148 [1.220, 3.779]	<.01	—					
Anemia	3.623 [2.316, 5.666]	<.0001	2.594 [1.625, 4.140]	<.0001				
Vascular disease^b^					3.288 [2.234, 4.838]	<.0001	2.085 [1.353, 3.212]	<.001
Antiplatelet agent use	2.226 [1.593, 3.112]	<.0001	2.005 [1.423, 2.824]	<.0001	2.796 [2.048, 3.817]	<.0001	1.670 [1.170, 2.383]	<.01
NSAIDs use	1.992 [0.980, 4.050]	.06	—					
P‐glycoprotein inhibitor^§^ use	1.481 [0.885, 2.477]	.13	—		1.200 [0.696, 2.069]	.51	—	

Abbreviations: CI, confidence interval; CLcr, creatinine clearance; HR, hazard ratio; NSAID, non‐steroidal anti‐inflammatory drug; TIA, transient ischemic attack.

^a^ See Table S1 for the definition of liver dysfunction used in the present study.

^b^ Myocardial infarction, internal carotid artery stenosis, or arteriosclerosis obliterans.

^c^ Quinidine, verapamil, erythromycin, or cyclosporine.

**TABLE 5:** Safety and effectiveness outcomes in subgroups of patients^a^

Variable	Safety outcomes: bleeding events		Effectiveness outcome
	All bleeding, n/N (%)	Major bleeding, n/N (%)	Ischemic stroke (excluding TIA) or systemic embolism, n/N (%)
All patients	808/9395 (8.60)	154/9395 (1.64)	164/9355 (1.75)
Old age (≥80 years)	321/3127 (10.27)	70/3127 (2.24)	67/3114 (2.15)
Severely low body weight (<40 kg)	43/367 (11.72)	14/367 (3.81)	10/364 (2.75)
Creatinine clearance (<30 mL/min)	56/537 (10.43)	17/537 (3.17)	11/529 (2.08)
Prior bleeding	103/483 (21.33)	21/483 (4.35)	11/480 (2.29)
Old age and low body weight (≥80 years and <40 kg)	34/261 (13.03)	11/261 (4.21)	9/259 (3.47)
Old age and renal dysfunction (≥80 years and <30 mL/min)	46/459 (10.02)	13/459 (2.83)	10/451 (2.22)
Old age and prior bleeding (≥80 years)	36/148 (24.32)	11/148 (7.43)	4/146 (2.74)
Low body weight and renal dysfunction (<40 kg and <30 mL/min)	15/111 (13.51)	4/111 (3.60)	5/110 (4.55)

Abbreviation: TIA, transient ischemic attack.

^a^ Patients receiving the recommended standard dose (60 mg) or the recommended reduced dose (30 mg) were included in the analysis.

### 3.4 Effectiveness analysis

Regarding clinical events, their cumulative incidence increased over 2 years (Figure 3B). The annual incidence of ischemic stroke (excluding TIA) or systemic embolism, stroke (excluding TIA) or systemic embolism, major cardiovascular events, death, and myocardial infarction was 1.08%, 1.44%, 1.83%, 1.28%, and 0.12%, respectively, and the annual incidence of all events was 2.72% (Table 3). The annual incidence of ischemic stroke (excluding TIA) or systemic embolism recurrence was 1.03% in the group who received the recommended standard dose and 1.20% in those who received the recommended reduced dose.

Multivariate analysis identified history of ischemic stroke or TIA, vascular disease, and antiplatelet agent use as risk factors for ischemic stroke (excluding TIA) or systemic embolism (*P* < .05) (Table 4). In analyses by patient characteristics, incidence of ischemic stroke (excluding TIA) or systemic embolism showed a tendency to be higher in patients with low body weight (<40 kg) and low CLcr (<30 mL/min) (4.55%), and in low‐bodyweight patients and older patients (≥80 years) (3.47%), than in the total population (1.75%) (Table 5).

## 4 DISCUSSION

Previously, we reported the results of an interim analysis of 1‐year data from the ETNA‐AF‐Japan study, which were reassuring regarding the safety and effectiveness of edoxaban.^15^ Here, we report the results of our final analyses at the completion of the 2‐year study period, to show the safety and effectiveness of edoxaban under the real‐world conditions of clinical practice.

In Japanese patients with factors associated with high risk for bleeding, such as low body weight, edoxaban is generally administered at the low dose of 30 mg/day, in accordance with the dose reduction criteria. Most patients in this study received edoxaban at 30 mg/day. Therefore, we consider information concerning the lower dose to be particularly important. In the previous 3‐month and 1‐year interim analyses, we evaluated data from patients classified into two groups according to edoxaban dose: 30 and 60 mg/day.^14^, ^15^ However, the former included under‐dose patients (ie, those who received the lower dose although they did not meet the dose reduction criteria) and the latter included over‐dose patients (ie those who received the higher dose although they did not meet the dose reduction criteria). In this study, we evaluated data from patients classified into over‐dose, recommended standard dose (60 mg), recommended reduced dose (30 mg), and under‐dose groups.

The present analysis included data from many patients with factors associated with high risk for bleeding, including older age, low body weight, and renal dysfunction. Because the dosing criteria for edoxaban specify body weight ≤60 kg and renal dysfunction as factors warranting dose reduction, there was a greater proportion of patients with the high risk of bleeding in the recommended reduced dose (30 mg) group than in the recommended standard dose (60 mg) group (Table 1). Additionally, there was a greater proportion of patients with high bleeding risk in the under‐dose group than in the recommended standard dose group. We attribute this finding to patients without dose adjustment factors being more likely to receive under‐dose if they were of an advanced age or had a history of bleeding, both of which are risk factors for bleeding.

Regarding clinical outcomes, incidences of both bleeding events and clinical events were similar to or lower than in the overall results for ENGAGE AF‐TIMI 48 (major bleeding, 2.75%/year; stroke or systemic embolism, 1.18%/year) and the results for Japanese patients in that study (major bleeding, 3.38%/year; stroke or systemic embolism, 1.47%/year).^4^, ^19^ This observation would suggest that edoxaban is well tolerated by Japanese patients with NVAF treated in a real‐world setting over the long term (ie 2 years of treatment).

Moreover, incidences of both all bleeding and ischemic stroke (excluding TIA) or systemic embolism in patients receiving the recommended reduced dose (30 mg) were similar to those in patients receiving the recommended standard dose (60 mg). The incidence of major bleeding showed a tendency to be higher in the group receiving the recommended reduced dose (1.21%/year) than in the group receiving the recommended standard dose (0.75%/year). A possible explanation is the higher bleeding risk in the reduced dose group as opposed to the standard dose group. Similar findings were also reported for apixaban, dabigatran, and rivaroxaban.^20^, ^21^, ^22^, ^23^ Furthermore, the incidence of major bleeding in the recommended reduced dose was no higher than in ENGAGE AF‐TIMI 48 (2.75%/year). Judging from these findings, we have no major concerns regarding edoxaban's safety and effectiveness when prescribed at the lower dose.

Despite prior concerns that bleeding events and clinical events would be more common in the over‐dose group and the under‐dose group, respectively, the results showed no such tendency. However, it should be noted that the data obtained in this study were not adjusted for patient background risk factors. Moreover, although patients were receiving the over‐dose or under‐dose at the start of treatment, the dose may have been adjusted eventually to the recommended dose following changes in factors such as body weight and renal function. Regarding the findings in the under‐dose group, although similar results have been reported,^24^ the results of other studies have shown a higher incidence of clinical events.^25^ Further studies are necessary to clarify the reasons for this discrepancy.

The multivariate analysis results showed that body weight, CLcr, history of gastrointestinal bleeding, anemia, and antiplatelet agent use were factors associated with major bleeding, and that history of ischemic stroke or TIA, vascular disease, and antiplatelet agent use were factors associated with ischemic stroke (excluding TIA) or systemic embolism. Most factors identified as risks for major bleeding were matched to those already estimated by the HAS‐BLED score,^16^ and those identified as risk factors for ischemic stroke (excluding TIA) or systemic embolism were almost matched to those already estimated by the CHADS_2_ and CHA_2_DS_2_‐VASc scores.^17^, ^18^ Lower body weight has previously been reported as a factor associated with major bleeding.^23^ However, no significant difference was found between patients with body weight <40 kg and those with ≥60 kg. Antiplatelet agent use may have been identified as one of the risk factors for ischemic stroke (excluding TIA) or systemic embolism because these drugs were likely to be prescribed to patients with complications and at high risk of thromboembolism.

In this study, we also evaluated edoxaban's safety and effectiveness in patients with high‐risk factors that are considered particularly important (ie age, body weight, renal function, and history of bleeding). Although these patients may not have been sufficiently included in pivotal clinical trials, physicians often encounter such patients in clinical practice, therefore the information is important. We found that the incidence of bleeding events was particularly high in patients aged ≥80 years and with a history of bleeding, and the incidence of ischemic stroke (excluding TIA) or systemic embolism was high in those with body weight <40 kg and CLcr <30 mL/min (Table 5). Considering the results of the multivariate analysis and edoxaban's safety and effectiveness profile in patient subgroups, anticoagulation should prompt careful monitoring to detect bleeding in patients thought to be at particularly high risk of this AE.

### 4.1 Limitations

This study has limitations. First, the nature of the study (an open‐label observational study) meant that it had no comparative arm. Therefore, a comparison of the safety and effectiveness of edoxaban with those of other DOACs or a vitamin K anticoagulant (eg warfarin) is not possible. Second, the results are based on data collected from case report forms completed by physicians, and data were missing from the forms of patients who failed to attend follow‐up visits. Consequently, event rates may be underestimated due to loss to follow‐up. Third, the analyses by doses were conducted based on dosing status at the treatment start, and changes to the doses and dose adjustment factors during the 2‐year follow‐up period were not considered.

## 5 CONCLUSIONS

The results obtained from this large‐scale study (>11 000 patients) confirm the good safety and effectiveness profile of edoxaban used over the long term (2 years) by Japanese patients with NVAF. No major concerns arose regarding the safety and effectiveness of the recommended reduced dose (30 mg), which patients with high bleeding risk are most likely to be prescribed.

## CONFLICT OF INTEREST

TY and YK have received remuneration from Bayer Yakuhin Ltd., Boehringer Ingelheim Japan Co. Ltd., Bristol Myers Squibb, and Daiichi Sankyo Co. Ltd. for consulting and lecturing services. TY has additionally received remuneration from Ono Pharmaceutical Co. Ltd. and Toa Eiyo Ltd. for consulting and lecturing services, and research funding from Bayer Yakuhin Ltd., Bristol Myers Squibb, and Daiichi Sankyo Co. Ltd. Daiichi Sankyo Co. Ltd. employs TN and KS. The In‐House Committee of Daiichi Sankyo Co., Ltd. and the Ministry of Health, Labour and Welfare of Japan (8 November 2014) approved the study protocol.

## DISCLOSURE

ETNA‐AF‐Japan was carried out in accordance with the Good Post‐marketing Study Practice of the MHLW. The study protocol was submitted to the MHLW after its approval by the relevant committee at Daiichi Sankyo Co. Ltd. All study participants provided informed consent. The study is registered with the UMIN Clinical Trials Registry (trial no. UMIN000017011).

## DATA SHARING AND DATA ACCESSIBILITY

Data from individual identified participants will not be shared. This includes information held in data dictionaries.

## Supporting information

Supplementary Material
