# Supplementary material for: Safety and effectiveness of edoxaban in Japanese patients with nonvalvular atrial fibrillation: Final report of a two‐year postmarketing surveillance study (ETNA‐AF‐Japan)
Source: J Arrhythm. 2021 Feb 24;37(2):370–83. doi: 10.1002/joa3.12520 (PMC8021991; doi:10.1002/joa3.12520)
Supplement: Supplementary file 1 — Supplementary Material [file JOA3-37-370-s001.docx]

# Supporting information

**Supplementary table 1.** Definition of liver dysfunction in the present study

| ALT | AST | | | |
| --- | --- | --- | --- | --- |
|  | <50 IU/L | ≥50 IU/L, <100 IU/L | ≥100 IU/L, <500 IU/L | ≥500 IU/L |
| <50 IU/L | Normal | Mild | Moderate | Severe |
| ≥50 IU/L, <100 IU/L | Mild | Mild | Moderate | Severe |
| ≥100 IU/L, <500 IU/L | Moderate | Moderate | Moderate | Severe |
| ≥500 IU/L | Severe | Severe | Severe | Severe |

ALT: alanine aminotransferase; AST: aspartate aminotransferase

**Supplementary table 2.** Incidence of major bleeding events in patients who received edoxaban (data from the safety analysis set, N = 11 111)^a^

| Event | n (%) |
| --- | --- |
| Total no. of patients, n (%) | 173 (1.56) |
| Total no. of events | 183 |
| Neoplasms benign, malignant and unspecified (incl cysts and polyps) | 3 (0.03) |
| Intracranial tumour haemorrhage | 1 (0.01) |
| Tumour haemorrhage | 2 (0.02) |
| Blood and lymphatic system disorders | 2 (0.02) |
| Anaemia | 1 (0.01) |
| Iron deficiency anaemia | 1 (0.01) |
| Acquired haemophilia | 0 (0.00) |
| Haemorrhagic diathesis | 0 (0.00) |
| Nervous system disorders | 57 (0.51) |
| Brain stem haemorrhage | 1 (0.01) |
| Cerebellar haemorrhage | 7 (0.06) |
| Cerebral haemorrhage | 24 (0.22) |
| Haemorrhage intracranial | 1 (0.01) |
| Intraventricular haemorrhage | 1 (0.01) |
| Subarachnoid haemorrhage | 10 (0.09) |
| Thalamus haemorrhage | 5 (0.05) |
| Putamen haemorrhage | 8 (0.07) |
| Intracranial haematoma | 1 (0.01) |
| Eye disorders | 2 (0.02) |
| Retinal haemorrhage | 2 (0.02) |
| Cardiac disorders | 4 (0.04) |
| Cardiac tamponade | 4 (0.04) |
| Pericardial haemorrhage | 0 (0.00) |
| Vascular disorders | 6 (0.05) |
| Aortic aneurysm rupture | 5 (0.05) |
| Haematoma | 1 (0.01) |
| Respiratory, thoracic and mediastinal disorders | 3 (0.03) |
| Epistaxis | 1 (0.01) |
| Pulmonary alveolar haemorrhage | 2 (0.02) |
| Gastrointestinal disorders | 80 (0.72) |
| Diverticulum intestinal haemorrhagic | 4 (0.04) |
| Duodenal ulcer haemorrhage | 1 (0.01) |
| Enterocolitis haemorrhagic | 1 (0.01) |
| Faeces discoloured | 1 (0.01) |
| Gastric haemorrhage | 9 (0.08) |
| Gastric ulcer haemorrhage | 5 (0.05) |
| Gastritis haemorrhagic | 1 (0.01) |
| Gastrointestinal haemorrhage | 27 (0.24) |
| Haematochezia | 1 (0.01) |
| Melaena | 5 (0.05) |
| Oesophageal varices haemorrhage | 2 (0.02) |
| Rectal haemorrhage | 1 (0.01) |
| Upper gastrointestinal haemorrhage | 5 (0.05) |
| Anal haemorrhage | 1 (0.01) |
| Lower gastrointestinal haemorrhage | 8 (0.07) |
| Large intestinal haemorrhage | 3 (0.03) |
| Small intestinal haemorrhage | 4 (0.04) |
| Haemorrhoidal haemorrhage | 2 (0.02) |
| Gastrointestinal mucosal disorder | 0 (0.00) |
| Abdominal wall haematoma | 1 (0.01) |
| Gastrointestinal polyp haemorrhage | 1 (0.01) |
| Gastrointestinal angiectasia | 1 (0.01) |
| Skin and subcutaneous tissue disorders | 1 (0.01) |
| Haemorrhage subcutaneous | 1 (0.01) |
| Musculoskeletal and connective tissue disorders | 4 (0.04) |
| Haemarthrosis | 2 (0.02) |
| Muscle haemorrhage | 2 (0.02) |
| Renal and urinary disorders | 1 (0.01) |
| Renal haemorrhage | 1 (0.01) |
| Injury, poisoning and procedural complications | 15 (0.14) |
| Subdural haematoma | 9 (0.08) |
| Subdural haemorrhage | 3 (0.03) |
| Post procedural haemorrhage | 1 (0.01) |
| Procedural haemorrhage | 1 (0.01) |
| Subarachnoid haematoma | 1 (0.01) |

^a^System organ classes were used in the calculation of the number of patients with AEs, and preferred terms were used in the calculation of the number of AEs (MedDRA version 22.1).
